# COVID-19 and psychological distress in Norway: The role of trust in the healthcare system

**DOI:** 10.1177/1403494820971512

**Published:** 2020-11-29

**Authors:** Samantha M. Harris, Gro M. Sandal

**Affiliations:** Department of Psychosocial Science, University of Bergen, Norway

**Keywords:** COVID-19, pandemics, health system trust, risk factors, corona, quarantine, social isolation, psychological distress, depression, anxiety, Norway

## Abstract

**Aim:**

The study aims to examine groups at risk for psychological distress in connection with the COVID-19 outbreak, and the role of trust in the healthcare system as a possible moderator.

**Methods:**

Data were collected from a large sample of the Norwegian population (*n* = 4008) through the Norwegian Citizen Panel (NCP). A linear regression was conducted to examine the effects of COVID-19 related risk factors on psychological distress, using the 10-item Hopkins Symptom Checklist (HSCL-10). Finally, we conducted a moderation analysis to examine the interaction of trust in the healthcare system and COVID-19 related risk factors.

**Results:**

A linear regression showed that female gender, younger age, lower level of education, being infected with COVID-19, being medically vulnerable, working in the healthcare system, being in voluntary quarantine and having an immigrant background predicted mean HSCL-10 scores. The moderation analysis revealed that people in the medically vulnerable group, those below 61, and those in quarantine reported higher psychological distress when they also had lower trust in the healthcare system.

**Conclusions:**

Findings indicate important groups to take into consideration in mental healthcare strategies and policies. However, most participants in the current study reported psychological distress levels that were below the clinical cut-off, suggesting that the majority may have coped relatively well in the early stages of the pandemic.

## Background

In connection with the ongoing COVID-19 outbreak, it is important to gather information about groups that are vulnerable to experiencing psychological distress. This will help us identify those most in need for mental health services. In this paper we examine the psychological distress correlates of belonging to vulnerable groups related to COVID-19 as well as the role of trust in the healthcare system as a potential moderator.

The Norwegian Institute of Public Health (NIPH) emphasizes that medically vulnerable people (e.g. the elderly and those with underlying diseases), and persons living in socially and economically vulnerable situations (e.g. low levels of education, having an immigrant background), as well as men, are at higher risk of contracting COVID-19 and experiencing negative-health related outcomes as well as COVID-19 related death [1]. Apart from smaller scale research [2] there is little empirical evidence about psychological health correlates of belonging to these vulnerable, demographic groups during the current pandemic.

Similarly, health professionals (HPs) constitute a vulnerable group, given their higher risk of infection [3], and their chance of experiencing potentially distressing events while caring for COVID-19 patients [4]. It has previously been suggested that HPs experience higher levels of psychological distress during an epidemic, due to the stigma associated with possibly carrying the disease in question [5]. Furthermore, a study from China suggested that HPs had a high prevalence of anxiety and depressive symptoms during the current pandemic [4]. However, there might be national differences in HPs psychological reactions due to differences in burdens on the healthcare system, access to human and material resources, and organizational factors. To date, there are few studies examining the psychological distress of HPs during the COVID-19 pandemic in a Scandinavian context [6].

To limit the spread of the virus, the Norwegian government has introduced strict measures including imposing mandatory quarantine and social distancing rules. Vulnerable groups, in particular, are advised to take precautions by social shielding in periods with widespread transmission in their community (voluntary quarantine). While these measures aim to protect the population, a review of studies conducted outside of Norway [6] reported that people experienced negative psychological effects in reaction to such measures, which may be long lasting. Again, these studies may not translate to a Scandinavian context, due to important societal differences linked to the social welfare system, food security, and income. Furthermore, quarantine may be experienced differently by people according to whether it is mandatory or voluntary [6]. This highlights the need to examine the psychological effects of both voluntary and mandatory quarantine in Norway.

Finally, we are interested in whether trust in the healthcare system could buffer potential adverse psychological consequences. During the current crisis, many people, particularly those with COVID-19 infections or those in vulnerable groups, rely on the healthcare system. Trust in the healthcare system in Norway is relatively high (77% [7] compared to an average of 40% across OECD countries [8]). A high level of trust has been associated with a range of positive health outcomes prior to the outbreak of COVID-19 [9,10]. Furthermore, populations with higher trust in the authorities are more likely to follow risk minimizing measures during pandemics [11]. On the other hand, mistrust in the healthcare system is associated with an increased likelihood of psychological distress [12]. It is unclear how mistrust in the healthcare system affects those in vulnerable situations during the pandemic, who may be discouraged from seeking help until their health condition deteriorates [12].

In summary, the current study will explore the effects of a range of demographic and COVID-19 related factors (hereafter referred to as ‘risk factors’) for psychological distress in a large sample of the Norwegian population during the weeks following the country’s lockdown, and the extent to which these are moderated by trust in the healthcare system.

## Methods

### Norwegian Citizen Panel

Data were collected through the Norwegian Citizen Panel (NCP) [13]. The NCP is a platform for internet-based surveys of public opinion regarding important areas of society and politics in Norway. Participants (18 and over) have been randomly selected from the national population register and the same group is invited repeatedly to participate. The current study was conducted as a ‘fast track’ survey during the first phase of the COVID-19 pandemic in Norway, March 2020. A total of 4008 participants were included in the final analyses. The invitation to take part in the survey was distributed to participants via email on the 20 March 2020. Two e-mail reminders were sent out on the 25 and 27 March. This was approximately one week after the implementation of strict infection control measures, including rules for quarantine.

### Hopkins Symptom Checklist (HSCL-10)

A Norwegian translation of the 10-item Hopkins Symptom Checklist (HSCL-10) [14,15] was used to measure depression- and anxiety-related symptoms over the last 7 days. The HSCL is a widely used measure in population surveys with high reliability and validity [16,17]. We used mean HSCL-10 scores in our analyses, calculated from a sum-score of the 10 items. The respondent indicates the relevance of each item on a four-point scale ranging from 1 = ‘not at all’ to 4 = ‘very much’. The clinical cut-off for psychological distress is ⩾ 1.85 [18]. Cronbach’s alpha in the present study was 0.87.

### Risk factors

Risk factors were self-reported and included the following: being medically vulnerable, age group, age above 61 (dichotomous), gender, being infected with COVID-19 (confirmed or suspected), level of education, and immigrant background (having migrated yourself, or being born to one or two migrant parents). The latter two factors relate to being socially/economically vulnerable [1]. Further, the survey assessed if respondents had been quarantined (mandatory or voluntary) and their occupation (working in the healthcare system, another critical occupation, or neither). Finally, trust in the healthcare system was assessed. Response categories are displayed in Table I.

**Table I.** Sample characteristics and differences in mean HSCL-10 scores, with significant post-hoc results.

Characteristics	*n*	%	Mean(SD)^a^	ANOVA^a^	Post-hoc comparisons^a^	
**Date of birth**						
1959 or earlier	1707	42.59	1.29(0.37)	F(2,3979) = 148.45, *p* < 0.001	*I vs II*	*p* < 0.001
1960-1989	1943	48.48	1.43(0.49)		II vs III	
1990 or later	358	8.93	1.66(0.54)		I vs III	
**Gender**						
Female	2042	50.95	1.49(0.50)	F(1,3980) = 38.65,*p* < 0.001		
Male	1966	49.05	1.40(0.48)			
**Highest level of education**						
No education/elementary school	208	5.19	1.52(0.51)	F(3,3978) = 15.28,*p* < 0.001	*I vs II*	*p* < 0.001
Secondary school	1233	30.76	1.41(0.51)		I vs III	
University	2498	62.33	1.41(0.43)			
Not answered	69	1.72				
**Immigration**						
Norwegian	3490	87.08	1.43(0.48)	F(4,3763) = 24.77,*p* < 0.001	*I vs II*	*p* < 0.001
I have immigrated myself	228	5.69	1.59(0.52)		I vs III	*p* < 0.001
Both parents have immigrated	16	0.40	2.15(0.86)		II vs III	*p* = 0.007
Mother has immigrated	68	1.70	1.47(0.54)		III vs IV	*p* = 0.001
Father has immigrated	56	1.40	1.59(0.56)		III vs V	*p* = 0.013
Not answered	150	3.74				
**Infected with COVID-19**						
Yes, confirmed by clinician or test	4	0.01	1.70(0.48)	F(3,3976) = 15.30,*p* < 0.001		
Yes, assumed	119	3.01	1.71(0.51)			
No, confirmed by clinician or test	90	2.25	1.53(0.54)			
No, assumed	3793	94.64	1.43(0.49)			
Not answered	2	0.00				
**In quarantine**						
Yes, mandatory	363	9.06	1.51(0.46)	F(2,3873) = 44.391, *p* < 0.001	*I vs II*	*p* = 0.004
Yes, voluntary	476	11.88	1.62(0.56)		II vs III	*p* < 0.001
No	3162	78.89	1.41(0.48)		I vs III	*p* < 0.001
Not answered	7	0.17				
**Medically vulnerable**						
Yes	1101	27.47	1.49 (0.52)	F(1,3956) = 11.05,*p* = 0.001		
No	2900	72.36	1.43 (0.48)			
Not answered	7	0.17				
**Trust in the healthcare system**						
Very high trust in the healthcare system	938	23.40	1.43(0.47)	F(6,3968) = 34.48, *p* < 0.001	I vs III	*p* = 0.024
High trust in the healthcare system	2294	57.24	1.38(0.42)		I vs IV	*p* < 0.001
Some trust in the healthcare system	430	10.73	1.52(0.54)		I vs V	*p* < 0.001
Neither trust nor mistrust in the healthcare system	102	2.54	1.72(0.56)		II vs III	*p* < 0.001
					II vs IV	*p* < 0.001
					II vs V	*p* < 0.001
Some mistrust in the healthcare system	100	2.50	1.81(0.73)		II vs VI	*p* = 0.010
High mistrust in the healthcare system	88	2.20	1.58(0.59)		II vs VII	*p* = 0.022
Very high mistrust in the healthcare system	47	1.17	1.71(0.77)		III vs IV	*p* = 0.022
Not answered	9	0.22			III vs V	*p* = 0.022
**Occupation critical to the pandemic**						
Healthcare service	403	10.05	1.42(0.43)	F(2,3964) = 0.789,*p* = 0.454		
Different critical function	504	12.57	1.43(0.46)			
No	3086	77.01	1.45(0.50)			
Not answered	15	0.37	-			

a Weighted by location, gender and age.

### Ethical considerations

All responses were anonymous, and data were stored and handled on a secure desktop (‘SAFE’), a solution for secure processing of sensitive personal data in research at the University of Bergen. Participation was voluntary and based on informed consent. The NCP follows all research ethics guidelines for the processing of information. The procedures for data collection and storage have been approved by the Norwegian Data Protection Authority.

### Statistical analyses

Statistical analyses were conducted using SPSS Statistics version 25 [19]. To examine differences in mean HSCL-10 scores we conducted one-way ANOVAs. We conducted Bonferroni post-hoc comparisons where variance was homogeneous, and Games Howell where variance was heterogeneous. To examine the extent to which variables jointly and individually predicted mean HSCL-10 scores, we conducted a multiple regression analysis. Risk factors were entered into model 1. Model 2 included risk factors as well as gender, age group, and highest completed education. We removed the variable for age above 61 from the analysis for model 2 to avoid collinearity with the age group variable.

Only four participants had both a confirmed coronavirus infection as well as having completed the HSCL-10. We, therefore, created a new dichotomous variable combining the participants that had either confirmed or assumed coronavirus infection into one category and those that had either confirmed or assumed non-infection into another. We also created dichotomous alternatives of the following variables: immigrant background (yes/no), trust in the healthcare system (high trust/low trust), quarantine (yes/no), and occupation (critical job/no critical job).

Furthermore, we conducted a moderation analysis to examine the effect of trust in the healthcare system (the moderator) on the effect of risk factors (independent variable) on mean HSCL-10 scores (dependent variable). This effect was examined by including the product of the independent variable and the moderator variable in the regression analysis. A significant interaction, therefore, indicates that the effect of the independent on the dependent variable depends on the moderator. The moderation analysis was conducted using the PROCESS macro for SPSS [20].

### Assumptions

There was linearity as assessed by partial regression plots and a plot of studentized residuals against the predicted values. Residuals were independent, as assessed by a Durbin–Watson statistic of 1.01 (model 1) and 1.13 (model 2). There was slight heteroskedasticity, as assessed by visual inspection of a plot of standardized residuals by standardized predicted values but given the small degree we decided to go ahead with the analysis. We found no evidence of multicollinearity, as assessed by tolerance values greater than 0.1, apart from age and the age above 61 variables. The latter was therefore removed from model 2. Several studentized deleted residuals were greater than ±3 standard deviations, however no leverage values greater than 0.2, and values for Cook’s distance above 1. Data were not normally distributed, however, we decided to go ahead with the analysis, since F-tests are generally accepted as being robust to non-normality [21].

### Variable weighting

Weights were applied to allow for valid statistical inferences and to compensate for bias. The weights are equal to the ratio of a given strata in the population to the total population, divided by the ratio of strata in the net sample to the total net sample. This procedure gives a value between 0 and 1. Respondents that are underrepresented receive a weight above 1, and respondents that are overrepresented receive a weight below 1. The weights are based on information about location, gender, and age from national register data. Weights were applied only to descriptive statistics (see Table I).

## Results

### Descriptive statistics

In total, 4008 participants completed the HSCL-10 and were included in the analyses. Post-hoc comparisons were not conducted for infection due to insufficient participants in the ‘yes, confirmed by clinician or test’ category. One-way ANOVAs revealed significant differences in mean HSCL-10 scores by all variables, apart from occupation (see Table I). Less than 1% reported a mean HSCL-10 score above the clinical cut-off. Sample characteristics (unweighted), along with mean HSCL-10 scores (weighted) and (significant) post-hoc results are presented in Table I.

### Multiple linear regression

#### Model 1

A multiple linear regression was conducted to predict mean HSCL-10 scores from the risk factors. The multiple regression model statistically significantly predicted mean HSCL-10 scores (F(9,3714) = 32.48, *p* < 0.001) . However, it is important to note that the adjusted *R*^2^ = 0.07, is generally seen as a very small effect size [22]. All variables, apart from working in a healthcare service and being in mandatory quarantine, added to the prediction. Regression coefficients and standard errors can be found in Table II.

**Table II.** Multiple regression results for mean HSCL-10 scores.

HSCL-10	*B*	95% CI for *B*		SE *B*	β	*R* ^2^	∆ *R*^2^
		LL	UL				
*Model 1*						0.07	0.07***
Constant	1.56***	1.50	1.62	0.03			
Infected with COVID-19	0.13**	0.05	0.22	0.02	0.05**		
Medically vulnerable	0.12***	0.10	0.15	0.02	0.13**		
Age above 61	–0.20***	–0.22	–0.16	0.02	–0.22***		
Trust in the healthcare system	–0.16***	–0.22	–0.12	0.03	–0.10***		
Quarantine							
Voluntary	0.07**	0.03	0.12	0.02	0.05**		
Mandatory	–0.02	–0.07	0.03	0.02	–0.01		
Occupation							
Healthcare service	–0.02	–0.07	0.02	0.02	–0.02		
Other critical capacity	–0.05*	–0.09	–0.05	0.02	–0.04*		
Immigrant background	0.11***	0.06	0.15	0.02	0.07***		
*Model 2*						0.12	0.12***
Constant	1.89***	1.80	2.00	0.05			
Female	0.15***	0.12	0.17	0.01	0.17***		
Age	–0.17***	–0.19	–0.15	–0.25	0.01***		
Education	–0.03**	–0.05	–0.01	0.01	–0.04**		
Infected with COVID-19	0.14**	0.06	0.22	0.04	–0.06**		
Medically vulnerable	0.13***	0.10	0.16	0.02	0.13***		
Trust in the healthcare system	–0.16***	–0.22	–0.11	0.03	–0.09***		
Quarantine							
Voluntary	0.05*	0.01	0.10	0.02	0.04*		
Mandatory	–0.03	–0.07	0.02	0.02	–0.02		
Occupation							
Healthcare service	–0.06*	–0.1	–0.01	0.02	–0.04*		
Other critical function	–0.04	–0.08	0.01	0.02	–0.02		
Immigrant background	0.11***	0.06	0.15	0.02	0.07***		

Model, ‘Enter’ method in SPSS Statistics; *B*, unstandardized regression coefficient; CI, confidence interval; LL, lower limit; UL, upper limit; SE *B*, standard error of the coefficient; β, standardized coefficient; *R*^2^, coefficient of determination; *∆ R*^2^, adjusted *R*^2^.

* *p* < 0.05, **p < 0.01, ***p < 0.001

Reference categories: male, not infected with COVID-19, not being medically vulnerable, age below 61, high trust in the healthcare system, and no immigrant background.

#### Model 2

We added gender, age group, and highest completed education to the analysis. The multiple regression model statistically significantly predicted mean HSCL-10 scores, *F*(11,3662) = 44.86, *p* < 0.001. The adjusted *R*^2^ = 0.12 increased in comparison to model 1 but is still generally seen as a small effect size [22]. Gender, age, and education contributed to the predictability of the model, *p* < 0.05. Following the inclusion of these variables, working in a critical capacity became non-significant, while working in the healthcare service became significant, in comparison to model 1 (Table II).

### Moderation analysis

We conducted a moderation analysis to examine the moderating effect of trust in the healthcare system (high trust/low trust) on risk factors and mean HSCL-10 scores.

**Figure 1.**
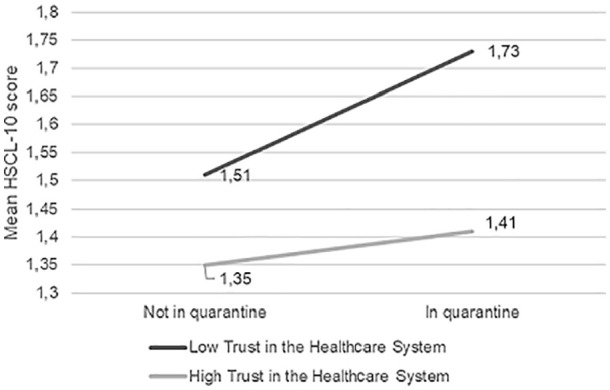
Moderation analysis of trust in the healthcare system as moderator between the effect of being in quarantine on mean HSCL-10 scores.

**Figure 2.**
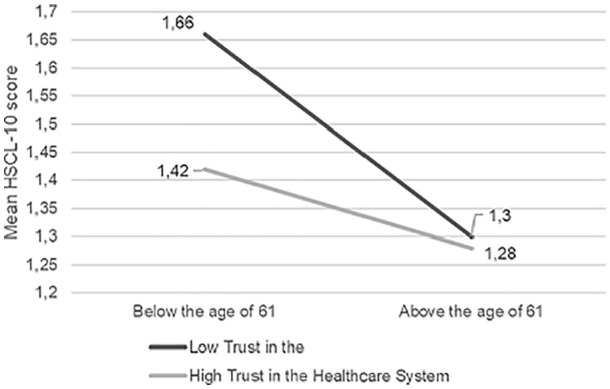
Moderation analysis of trust in the healthcare system as moderator between the effect of age on mean HSCL-10 scores.

**Figure 3.**
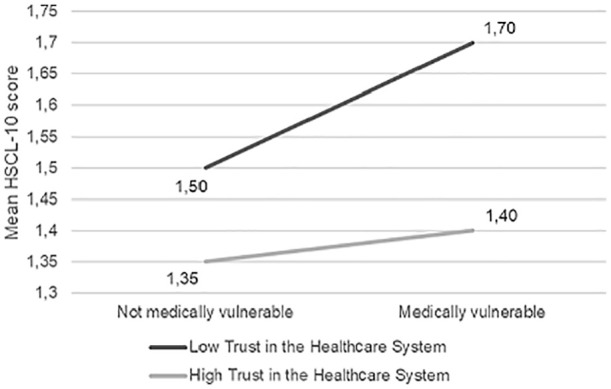
Moderation analysis of trust in the healthcare system as moderator between the effect of being medically vulnerable on mean HSCL-10 scores.

We found that 80% of participants reported high or very high trust in the healthcare system. Furthermore, the moderation analysis revealed that trust in the healthcare system interacted with several risk factors. There was an interaction effect of trust in the healthcare system and being medically vulnerable (*b* =−0.15, 95% confidence interval (CI) (−0.29, −0.02), *t* = −2.30, *p* = 0.021). Upon examining the simple effects (see Figure 1), it appeared that people in the medically vulnerable group were more likely to report higher psychological distress if they also reported lower trust in the healthcare system (conditional effect = 0.20, 95% CI (0.07, 0.32), *t* = 3.09, *p* = 0.002) than those with high trust in the healthcare system (conditional effect = 0.05, 95% CI (0.00, 0.08), *t* = 2.83, *p* = 0.005). There was an interaction effect of age above 61 and trust in the healthcare system (*b* = 0.22,9 5% CI (0.10, 0.34), *t* = 3,56, *p* < 0.001), in that participants below 61 seemed to be more affected by low trust in the healthcare system (conditional effect = −0.24, 95% CI (−0.31,−0.17), *t* = −7.02, *p* < 0.001) than those above 61 (conditional effect = −0.02, 95% CI (−0.12, 0.8), *t* = −0.38, *p* = 0.707) (Figure 2). Similarly, there was an interaction effect of trust and being in quarantine (mandatory and voluntary combined) (*b* = −0.16, 95% CI (−0.29, −0.02), *t* = −2.30, *p* = 0.021), in that people in quarantine that also had low trust in the healthcare system were more likely to report higher psychological distress (conditional effect = 0.22, 95% CI (0.86, 0.35), *t* = 3.25, *p* = 0.001) than those not in quarantine (conditional effect = 0.06, 95% CI (0.03, 0.09), *t* = 3.26, *p* = 0.001) (Figure 3).

There was no interaction effect of trust and occupation (*b* = 0.13, 95% CI(–0.00, 0.26), *t* = 1.90, *p* = 0.057), infection with coronavirus (*b* = 0.021, 95% CI (–0.05, 0.46), *t* = 1.59, *p* = 0.112), or immigrant background (*b* = 0.01, 95% CI(–0.14, 0.16), *t* = 0.13, *p* = 0.985).

## Discussion

The present study suggests that belonging to certain vulnerable groups related to COVID-19 is associated with more psychological distress. The multiple regression (model 2) suggested that factors such as being medically vulnerable, being an immigrant, having a lower level of attained education, working as an HP, as well as having been infected with the virus predicted higher HSCL-10 scores. However, the findings also suggest that not all types of risk are associated with psychological distress. Regarding gender and age, for example, those with the highest risk (males and elderly) indicated lowest distress. It should be noted that the total explained variance was limited to 12%.

Trust in the healthcare system appears to have risen during the current pandemic [23]. However, the moderation analysis revealed that lower trust in the healthcare system was related to higher psychological distress in some groups. While these effects were modest and causal relationships cannot be established, the results are consistent with past research [12] and suggest that building trust in the healthcare system may be particularly beneficial for these groups during the pandemic.

Contrary to previous studies, which show that HPs report higher levels of psychological distress [6], the first step of our analysis (model 1) suggested that their reported distress did not differ from other participants (model 1). However, once we added age, gender and education to the analysis (model 2), findings became significant. This suppressor effect suggests that the effect of working as an HP is ‘hidden’ by the effect of these demographic variables and that HPs may in fact be experiencing higher psychological distress. These findings add weight to calls for supporting healthcare workers during and following the current pandemic [3]. Furthermore, the fact that working in another capacity (‘other critical function’) attenuated, suggests that age, gender and education explain some of its effect.

In addition, our results suggest that people who quarantined voluntarily reported higher psychological distress than people who were in mandatory quarantine, which is inconsistent with previous research [6]. People with pre-existing anxiety may be more likely to self-isolate voluntarily during the current pandemic [24], meaning higher psychological distress may have pre-dated quarantine in our study, rather than vice versa. However, due to the cross-sectional design of our study, we are unable to draw conclusions regarding causal effects. It is important to note that social shielding may present an additional burden to people belonging to risk groups, due to the lack of social support that could protect against stress caused by the pandemic [25].

Past research suggests that prolonged quarantine causes greater detriments to mental well-being [6]. Data collection for the current study was conducted 1–2 weeks following the lockdown, suggesting that participants had probably spent a maximum of 2 weeks in quarantine. Our findings as well as the relatively small effect sizes may be partly due to the short amount of time participants spent in quarantine.

Older people, who are physically more vulnerable to the virus, reported lower psychological distress. This finding is mirrored in another COVID-19 study [2], as well as pre-COVID research [26]. While the young generation are at lower risk for experiencing negative physical consequences from COVID-19, they may be more strongly affected by financial uncertainty and governmental measures including the closing of universities, childcare centres, and schools. This study should be considered in light of certain strengths and limitations. An asset of the study is that it includes a large sample of the Norwegian population, and by applying the variable weights we ensured a good level of representativeness in the descriptive data. Furthermore, this is one of the first studies to examine the role of trust in the healthcare system for psychological distress during the COVID-19 pandemic.

However, we also recognize limitations. First, combining categories into dichotomous variables, such as combining people with suspected and confirmed virus into one group, may have led to the loss of information as well as introduced bias. Similarly, trust in the healthcare system was only measured by one item. Secondly, the study was cross-sectional and conclusions on the causes of psychological distress cannot be made. Some of the groups that reported higher psychological distress, for example, are also known to report higher psychological distress prior to the pandemic (e.g. women [27] and migrants [28]). Furthermore, other potential risk factors, such as financial strain and job insecurity, were not considered in our survey. Thirdly, the data did not perfectly meet assumptions for a regression analysis. While violating assumptions does not necessarily bias the coefficient estimates, it may make them less precise, and may increase the risk of type 2 errors. Finally, despite a substantial sample size, the 4008 participants included in our study were not completely representative of the Norwegian population. For example, those born after 1990 were under-represented, while those born before 1959 were over-represented [29]. This may have introduced bias in the analyses, where the variable weightings were not applied. Furthermore, having conducted the survey online may have led to a skewed sample, for example regarding elderly participants who might be less skilled in digital platforms than younger age groups.

## Conclusion

This survey study indicates that certain groups in the population were more likely to experience psychological distress during the first weeks following the COVID-19 lockdown in Norway and revealed the moderating effect of trust in the healthcare system. These findings are important from a policy perspective and could inform mental health care strategies to target vulnerable groups during pandemics. The relatively small effect sizes suggest that much of the population may not have experienced high levels of psychological distress during the first weeks of the lockdown. Longitudinal studies are needed to delineate the long-term effects of the pandemic on peoples’ psychological wellbeing as well as directions of causalities.
